# Prediction of Mortality in Patients on Peritoneal Dialysis Based on the Fibrinogen Mannosylation

**DOI:** 10.3390/cells12030351

**Published:** 2023-01-17

**Authors:** Marko Baralić, Lucia Pažitná, Voin Brković, Mirjana Laušević, Nikola Gligorijević, Jaroslav Katrlík, Olgica Nedić, Dragana Robajac

**Affiliations:** 1 School of Medicine, University of Belgrade, 11000 Belgrade, Serbia; 2 Clinic of Nephrology, University Clinical Centre of Serbia, 11000 Belgrade, Serbia; 3 Institute of Chemistry, Slovak Academy of Sciences, 84538 Bratislava, Slovakia; 4 Institute for the Application of Nuclear Energy (INEP), University of Belgrade, 11080 Belgrade, Serbia

**Keywords:** ESRD, CKD, glycosylation, N-glycans

## Abstract

As we already reported, fibrinogen fucosylation emerged as a prognostic marker of peritoneal membrane function in end-stage renal disease (ESRD) patients on peritoneal dialysis. After a follow-up period of 18 months, we estimated the ability of employed lectins, as well as other biochemical parameters, to serve as mortality predictors in these patients. Following a univariate Cox regression analysis, ferritin, urea clearance, residual diuresis, hyperglycemia, and an increase in the signal intensity obtained with Galanthus nivalis lectin (GNL) emerged as potential mortality predictors, but additional multivariate Cox regression analysis pointed only to glucose concentration and GNL as mortality predictors. Higher signal intensity obtained with GNL in patients that died suggested the importance of paucimannosidic/highly mannosidic N-glycan structures on fibrinogen as factors that are related to unwanted cardiovascular events and all-cause mortality and can possibly be seen as a prediction tool. Altered glycan structures composed of mannose residues are expected to affect the reactivity of mannosylated glycoproteins with mannose-binding lectin and possibly the entire cascade of events linked to this lectin. Since patients with ESRD are prone to cardiovascular complications and the formation of atherosclerotic plaques, one can hypothesize that fibrinogen with increasingly exposed mannose residues may contribute to the unwanted events.

## 1. Introduction

Fibrinogen is a 340 kDa glycoprotein built of two sets of three polypeptide chains in form (AαBβγ)_2_, synthesized in hepatocytes. It is susceptible to various post-translational modifications, such as oxidation, nitration, acetylation, carbamylation, guanidinylation, phosphorylation, glycation, and glycosylation [1]. Fibrinogen is involved in hemostasis, and its coagulation properties can be affected by structural modifications or changes in its concentration. Glycosylation is a co/post-translational modification that can significantly alter functions and interactions of glycobiomolecules, affecting crucial processes such as cell adhesion, migration, and signal transduction [2]. It is postulated that approximately 60% of all tissue proteins are N-glycosylated, i.e., carbohydrates are covalently bound to Asn residue in a sequence Asn-X-Ser/Thr, where X can be any amino acid except Pro [3]. There are three types of N-glycans: high mannose, hybrid and complex. All three fibrinogen chains can be N-glycosylated: Aα chain at positions Asn453 and Asn686, Bβ chain at position Asn394, and γ chain at Asn78 [4,5]. Glycosylation of fibrinogen in different conditions is a relatively novel and unexplored topic, with only a few papers reported so far regarding pregnancy [6], aging [7,8], cirrhosis [9], hepatocellular carcinoma [10] and end-stage renal disease-ESRD [11]. 

Increased fibrinogen concentrations are related to an increased risk of cardiovascular and all-cause mortality rates in patients undergoing peritoneal dialysis [12]. It was demonstrated that glycosylation affects the rate of its polymerization and fiber thickness [7]. In our previous study, we discussed altered glycosylation of fibrinogen in patients with ESRD on peritoneal dialysis and proposed fibrinogen fucosylation as a prognostic marker of peritoneal membrane damage. After a follow-up period of 18 months, our investigation targeted the potential application of fibrinogen carbohydrates, biochemical and dialysis parameters, and the primary cause of ESRD as predictors of all-cause and cardiovascular mortality in the same group of patients.

## 2. Materials and Methods

### 2.1. Study Group

Patients with ESRD on continuous ambulatory peritoneal dialysis (*n* = 52), treated at the Clinics of Nephrology of the University Clinical Centre of Serbia (UCCS), were recruited in December 2019 and followed up in a prospective study for 18 months. The study was approved by the Ethical Committee of UCCS and INEP (no. 890/8, from 21.12.2018.). All participants gave written consent, and the study was performed in accordance with the Declaration of Helsinki. The study included only patients who, in the period of three months before sample collection, did not have peritonitis nor clinical and laboratory signs of the infection of the exit site (place of the peritoneal catheter). Patients who were taking oral anticoagulants and/or antiplatelet therapy were excluded from the study, as well as patients who previously had known coagulopathy or hematological malignancy. Patients with acute or chronic liver lesions were also excluded, which meant that all examined patients had a negative virological status for hepatotropic viruses (anti-HCV and HbsAg), and in biochemical analyzes, they had aminotransferases (AST and ALT), gamma-GT, and bilirubin (direct and indirect) within reference values.

### 2.2. Samples

Sampling was performed at the beginning of the study. Serum and plasma samples were collected early in the morning for hematological (Beckman Coulter® HmX Hematology Analyzer, Beckman Coulter, Inc., Brea, CA, USA) and biochemical analysis (Architect ci8200, Abbott Diagnostics, Wiesbaden, Germany), as well as for fibrinogen isolation. Detailed information and results are given in Baralić et al. [11].

### 2.3. Fibrinogen Isolation and Glycoanalysis

Fibrinogen was isolated from 500 μL of plasma using ammonium sulfate solution in a final concentration of 20% [11]. The precipitate was separated by centrifugation at 10,000× *g* for 5 min and dissolved in 50 mM phosphate buffer containing 150 mM sodium chloride (PBS, pH 7.4). Fibrinogen concentration was further adjusted to 100 μg/mL using PBS, and all samples were analyzed by lectin-based protein microarray [13]. Briefly, fibrinogen samples were printed on epoxysilane-coated microarray slides (NEXTERION Slide E, Schott, Germany) in eight identical subarrays using piezoelectric printer sciFLEXARRAYER S1 equipped with piezo dispense capillary PDC 80 (Scienion AG, Berlin, Germany), at a temperature of 14 °C and humidity of 60%. After incubation at 4 °C for 2 h, unoccupied reactive sites on slides were blocked with 3% bovine serum albumin in PBS at 4 °C for 1 h. The excess of the blocking agent was removed by washing, and the printed samples were incubated with biotinylated lectins (25 µg/mL in PBS with 0.05% Tween 20-PBST) at 25 °C for 1 h (the list of lectins is given in [11]). After thorough washing with PBS, bound lectins were left to interact with 0.5 µg/mL CF647-streptavidin conjugate in PBS at 25 °C for 15 min. Slides were then thoroughly washed with PBST and distilled water, dried by centrifugation, and scanned using InnoScan®710 fluorescent scanner (Innopsys, Carbonne, France). The obtained signals were analyzed using Mapix®7.4.1 software (Innopsys).

### 2.4. Peritoneal Membrane Function

Peritoneal Equilibration Test (PET) enables assessment of peritoneal membrane transport characteristics, where the rate at which solutes are transported through the peritoneal membrane is determined until equilibrium is established for a given substance on both sides of the peritoneal membrane in serum (circulation) and infused dialysis solution [14]. The test was performed according to the recommendations of Cnossen et al. [15].

### 2.5. Comorbidity Assessment

ICED (Index of Coexistent Disease) consists of two sub-indices: the IDS (the Index of Disease Severity) and the IPI (the Index of Physical Impairment). The IDS index was determined based on data from electronic medical history, while the determination of the IPI index was performed from the data obtained through an interview. The ICED index is calculated based on a combination of the IDS and IPI indices, according to Miskulin et al. [16]. The Charlson comorbidity index (CCI) was calculated at the beginning of the study using an online calculator [17].

### 2.6. Statistical Analysis

Following the Kolmogorov–Smirnov test of statistical normality, continuous variables are presented as mean ± SD. Univariate Cox proportional-hazards analysis was used to identify predictors of lethal outcomes in the follow-up period. Those variables that show significant prediction at a *p*-level less than 0.1 were included in the multivariate Cox model using a forward stepwise (likelihood ratio) method of entry. We considered as independent predictors of mortality those variables with a *p*-value less than 0.05. Statistical analysis was performed using the SPSS, version 18.0 (SPSS Inc., Chicago, IL, USA) software package.

## 3. Results

Basic demographic and clinical data on patients and dialysis procedures were given in the article of Baralić et al. [11]. At the beginning of the study, urea clearance in the entire investigated population was, on average, higher than 1.7, while weekly clearance of creatinine was higher than 60 L (Table 1).

More than 50% of patients involved in the study had some unwanted cardiovascular event before inclusion in the study: cardiovascular insult (11.5%), congestive heart failure (46.1%), or acute myocardial infarction (17.3%). Only one of these events occurred in 14 patients (26.9%). Two events affected 10 patients (19.2%), while all three events happened to two patients (3.8%). All patients with acute myocardial infarction had consequential congestive heart failure, and 4 out of 6 patients with cardiovascular insult were diagnosed with congestive heart failure.

Indexes for physical impairment, disease severity, and coexisting disease (IDS, IPI and ICED) for the investigated patient cohort were also investigated and are given in Table 2. Out of 52 patients, 36 had calculated the Charlson comorbidity index equal to or higher than 7.

Eighteen months after the beginning of the study, the survival rate of participants was calculated. Thirteen patients died (25%) due to a cardiovascular event: 12 due to ischemic heart disease and one due to ischemic cerebrovascular insult. The average age of diseased patients was 65.7 ± 14.3 years, and the majority were female (8 or 61.5% of diseased). The average age of patients transported to other renal replacement therapies (RRT) was 61.7 ± 23.2 years, and all of them were male: two patients were transferred to hemodialysis, and two received a cadaveric kidney transplant. Altogether after a follow-up period of 18 months, 35 patients (67.31%) successfully reached the end of the study.

Detailed results of the lectin microarray analysis of the isolated fibrinogen samples from patients (with 16 lectins) are shown in the work of Baralić et al. [11]. For the purpose of this study, these results were combined with all other data on patients collected during the 18-month follow-up period. Responses of all 16 lectins were examined in that context. Univariate Cox regression analysis was employed to relate mortality/morbidity rates and unwanted cardiovascular events with the results of lectin microarray. The reactivity of Galanthus nivalis lectin (GNL), specific for mannosylated structures, was found to be associated with some of the clinical indicators. The results of the regression analysis comparisons of data for survivors and non-survivors are given in Table 3. All variables relevant to dialysis efficiency were taken into consideration (outcome, demographic, clinical, and biochemical parameters).

A frequency of peritonitis events and comorbidities (represented by IPI and ICED indexes) emerged as the strongest mortality predictors besides ferritin concentration.

The primary cause which led to the development of ESRD was not the same for all patients, so the correlation between the cause and GNL response was examined. As shown in Table 4, the prevalence of diabetic nephropathy was the highest (30.7%), followed by angiosclerosis (25.0%) and glomerulonephropathy (21.1%). No correlation between disease cause and lectin reactivity was found (data are shown only for GNL).

Parameters that gave α value less than 10% in univariate Cox regression analysis (Table 3) were further subjected to multivariate Cox regression analysis, and results are shown in Table 5. No significant differences were found for the age, primary disease duration, peritonitis rate, dialysis performance, blood pressure, albumin concentration, and ferritin concentration, so these parameters were discarded as mortality prediction factors. On the other hand, patients that did not survive had significantly higher levels of glucose (*p* = 0.037) and GNL signal (*p* = 0.010).

## 4. Discussion

In our previous study, we reported on fibrinogen glycosylation in ESRD patients on peritoneal dialysis, emphasizing the importance of fucosylation as a prognostic marker of peritoneal membrane function [11]. After an 18-month period of follow-up, we estimated the potential of lectins to serve as mortality predictors in these patients. Higher signal intensity for GNL in patients that died suggested the importance of paucimannosidic/highly mannosidic N-glycan structures on fibrinogen as factors that are related to unwanted cardiovascular events and can possibly be seen as a prediction tool.

Some ESRD patients are more prone to hemorrhage, whereas others suffer from thrombosis [18]. Major unwanted cardiovascular events are leading causes of mortality in patients on hemodialysis [19]. Although less commonly applied, peritoneal dialysis is associated with a 48% lower mortality rate compared to hemodialysis in the first 2 years of RRT [20]. Charlson comorbidity index is commonly used as a predictive factor of mortality in a 10-year period [21,22]. Observed average CCI of 7.62 and 32 patients (61.5%) with an index equal to or higher than 7 enabled a prediction that in a 10-year period, 60% of them are at extremely high risk of unwanted cardiovascular event with lethal outcomes. The ICED index also indicates the significant disease severity and presence of physical limitations. These findings can somewhat be explained by the age of the patients, the dominant selection of patients with cardiovascular diseases for peritoneal dialysis, the high prevalence of diabetic patients as well as the fact that the research was conducted in a tertiary center where most patients are with comorbidities that require the application of more complex procedures. 

After a follow-up period of 18 months, four patients (all male) were transferred to another RRT method, whereas 13 patients died (mostly female), which is not in accordance with a long-term observation by Kitterer et al. [23] that women have a higher survival rate. In our study group, the most prevalent primary disease was diabetic nephropathy (30.7%), followed by nephroangiosclerosis (25.0%). According to the values obtained in PET, patients were slow transporters for glucose and slow-average for creatinine [24], which are indicative parameters for the application of continuous ambulatory peritoneal dialysis. In addition, the mean value for Kt/V is 3.47, which indicates significant adequacy of dialysis. The recommendations indicate that the average UF should be optimized and individualized according to the patient in order to primarily achieve a euvolemic state [25]. The percentage of males who had more preserved diuresis was significantly higher than females, while in patients with longer dialysis experience, there was a decrease in the amount of RU, which is in accordance with the literature [26]. This also explains the lower mortality rate of male patients in the studied group. Although decreased residual renal function indicates increased mortality caused by some adverse event of CVD, the study groups had no statistical significance in terms of these parameters when compared with the group of patients who had a higher volume of RU. This result indicates the need for more careful monitoring of dialysis adequacy parameters, especially in certain subgroups of patients, such as those with reduced residual diuresis, as well as in patients with weaker glycoregulation and inflammation. On the other hand, testing for fibrinogen glycosylation as a potential predictor of mortality may be important in identifying patients who require more detailed cardiac monitoring, even if they do not have specific cardiac disorders.

After univariate Cox regression analysis, the following parameters emerged as potential mortality predictors: ferritin, urea clearance, residual diuresis, hyperglycemia, and an increase in GNL signal. Ferritin level was already reported as a mortality predictor [27], whereas other parameters, except glycans recognized by GNL lectin, are generally considered important for the outcome but are not termed as “predictors” of 1-year mortality [28]. Although age and albumin are expected to be important factors in mortality prediction, we found no correlation between these parameters and lethality outcome. This might be somewhat explained by the relatively small sample size. The mortality-associated prediction potential of GNL in relation to fibrinogen glycosylation emerged in the present study. So far, only sporadic information regarding protein glycosylation in peritoneal dialysis can be found [29]. The observed changes occur mostly due to morphological and functional alterations in a peritoneal membrane. Additional multivariate Cox regression analysis pointed only to glucose concentration and GNL signal intensity as potential predictive markers of all-cause mortality in our investigated group.

GNL is a homotetramer (50 kDa) belonging to the GNL-related lectin family; mannose-binding specific lectins have a broad spectrum of biological functions with therapeutical and diagnostic potential [30]. GNL recognizes high-mannose-type N-glycans and preferentially binds to terminal mannose residues but also shows a bit of cross-reactivity with terminal galactose residues [31]. This lectin was used for glycan analysis of various pathologies in many formats, including microarray [11,32,33,34]. Due to the absence of RCA reactivity [11], one might postulate that an increased content of high mannose (Man5-9GlcNAc2, also known as M5–M9) or paucimannosidic structures (Man1-4GlcNAc2, also known as M1–M4) on fibrinogen is a mortality predictor biomarker. High-mannose N-glycans are precursors of hybrid and complex N-glycans and can be perceived as intermediates in the glycosylation pathway, which is orchestrated by the action of different glycosydases (such as mannosidase) and glycosyl-transferases (such as N-acetylglucosaminyl-, galactosyl-, sialyl- and fucosyl-transferase). In the Golgi apparatus, high-mannose glycans are first trimmed by the action of α-mannosidase (encoded by MAN1A1) and further subjected to N-acetylglucosaminyl-transferase I (GnT-I, encoded by MGAT1), which is the first enzyme in the sequence of glycosyl-transferases, that adds β1,2-N-acetylglucosamine to the tri-mannose core, specifically α1,3-linked mannose arm [35,36]. Low expression of mentioned enzymes can lead to an increase in the content of high-mannose and/or paucimmanosidic structures or even incomplete synthesis of complex N-glycans. An increase in the content of high-mannose glycans is associated with dedifferentiation of well-differentiated human hepatocellular carcinoma tissue, while a low expression of MGAT1 in moderately-differentiated tumors is associated with intrahepatic metastasis and poor prognosis [37]. Extended high-mannose N-glycans promote metastasis of cholangiocarcinoma in mice membrane proteins via down-regulation of MAN1A1, as shown in MAN1A1-overexpressing KKU-213AL5 cells [38], while M6, M9, and A3F structures may be associated with tumor progression in human cholangiocarcinoma [39]. Low-abundant serum high-mannose N-glycans are increased in patients with breast cancer (specifically M9) and are correlated with the progression of breast cancer in mice [40]. Levels of paucimannosidic N-glycans (Man1-3Fuc0-1GlcNAc2) are increased in colorectal carcinoma tissue [41], and their high levels are correlated with poor prognosis [42]. IL-6 and progesterone can modulate the expression of oligosaccharyl-transferase, an enzyme that catalyzes the transfer of glycan from a lipid-linked oligosaccharide to Asn in Asn-X-Ser/Thr sequence, resulting in up to a 30% increase in high-mannose structures on IgG Fab region [43]. Therapeutic IgG1 and IgG2 antibodies containing higher levels of high-mannose glycans (M5) in the Fc region are cleared more rapidly than other glycan forms [44]. High-mannose-containing oligosaccharides significantly contribute to the interaction between coagulation factor VIII and lectin mannose-binding 1 (LMAN1), a transmembrane protein localized in the endoplasmic reticulum/Golgi apparatus intermediate compartment which is involved in the secretion of a subtype of glycoproteins [45]. When taking into account all said, the importance and contribution of (pauci)mannosidic N-glycans in the diagnosis and prognosis of diseases is undoubtful and is just starting to reveal its potential.

Modified glycans may further interfere with protein interactions and functions. Human plasma is a source of six lectins, differentiated into four families based on their structural and biochemical characteristics: pentraxins (C-reactive protein and serum amyloid protein), collectin (mannose or mannan-binding lectin-MBL), ficolins (H- and L-ficolin) and tetranectin [46]. Human lectins are mostly involved in immunity and inflammation. Local and systemic inflammation and oxidative stress are common in patients on peritoneal dialysis, and some of their parameters are recently being considered as prognostic/diagnostic markers of PD [47,48]. Lectin MBL, a component of the lectin complement pathway and one of the initiators of the immune response [49], was correlated with the immune-mediated rejection of liver and kidney allograft, cerebrovascular insult, and some gastrointestinal pathologies [50]. Increased levels of plasma and urine complement concentrations (including MBL) were found in patients with diabetic nephropathy, and complement activation (alternative and lectin pathway) correlated with the development and severity of renal damage [51,52]. MBL was identified as a contributor to the progression of diabetic nephropathy [51,52,53]. Although low MBL levels are associated with a higher risk of cardiac and cardiovascular events in patients on hemodialysis [54], the MBL2 genotype was not related to any long-term clinical effect and outcome in ESRD patients on peritoneal or hemodialysis or with a functional graft [55]. In some patients with IgA and Henoch-Schönlein nephropathy, depositions of MBL-IgA aggregates were observed [56]. Matthijsen et al. [57] demonstrated the involvement of increased local production of MBL in myeloid cells in early atherosclerosis, while Biezeveld et al. [58] demonstrated the involvement of MBL2 polymorphism in the development of coronary artery lesions in patients with Kawasaki disease.

Cardiovascular diseases are the leading cause of death in patients treated with methods of renal function replacement, and in a certain number of patients, there are no symptoms or signs of heart disease. Our data suggest that a periodical scan of fibrinogen mannosylation (signals obtained with GNL) might lead to earlier and timely discovery of potential cardiovascular problems. Upon additional diagnostics, such as a load test or elective coronary angiography, significant plaques could be detected. This could further require balloon dilatation or placement of stents, or if changes are extensive, the cardiosurgical intervention of myocardium revascularization.

## 5. Conclusions

Altered glycan structures composed of mannose residues are expected to affect the reactivity of mannosylated glycoproteins with MBL and possibly the entire cascade of events linked to this lectin. Since patients with ESRD are prone to cardiovascular complications and the formation of atherosclerotic plaques, one can hypothesize that fibrinogen with increasingly exposed mannose residues may contribute to the unwanted events. Although mechanisms underlying the involvement of modified fibrinogen in the plaque formation or initiation of MBL-associated pathways are unknown, results of this study unequivocally relate fibrinogen mannosylation and mortality rates in patients on peritoneal dialysis and indicate the need for better cardiovascular monitoring even in patients without specific cardiac problems.

## Figures and Tables

**Table 1:** Dialysis characteristics and dialysis efficiency in patients on peritoneal dialysis (*n* = 52).

	Mean	SD	Min–Max Value
Peritonitis rate prior to the beginning of study	0.58	0.94	0–4
Residual urine (L/day)	0.93	0.82	0–3.70
Ultrafiltration (L/day)	1.10	0.52	0.1–2.80
Clearance of urea (Kt/V)	3.47	0.53	1.38–4.07
Weekly clearance of creatinine (L/week)	78.65	19.77	49.14–118.70
Peritoneal equilibration test with glucose (PETgly)	0.46	0.10	0.21–0.67
Peritoneal equilibration test with creatinine (PETcr)	0.63	0.11	0.31–0.87

**Table 2:** Distribution of physical impairment indexes and the Charlson comorbidity index in patients on peritoneal dialysis (*n* = 52).

	Mean	SD	Min–Max Value
Index of physical impairment (IPI)	0.71	0.72	0–2
Index of disease severity (IDS)	2.25	0.79	1–3
Index of coexistent disease (ICED)	2.27	0.79	1–3
Charlson comorbidity index (CCI)	7.63	2.19	3–15

**Table 3:** Univariate Cox proportional-hazards analyses for predictors of lethal outcome. Survivors (*n* = 39) and non-survivors (*n* = 13). Significant differences are given in bold.

	Hazard Ratio	95% Confidence Interval		*p*-Value
		Lower	Upper	
Age	0.995	0.958	1.034	0.810
Albumin	0.977	0.859	1.111	0.722
Peritoneal equilibration test for creatinine (PETcr)	0.042	0.001	3.045	0.147
Peritoneal equilibration test for glucose (PETgly)	0.480	0.003	72.866	0.774
Prathyroid hormone (iPTH)	1.000	0.999	1.001	0.830
Urea clearance (Kt/V)	0.289	0.083	1.007	**0.051**
Weekly creatinine clearance (Ccr)	0.979	0.951	1.007	0.146
Glucose	1.098	0.985	1.224	**0.093**
Ferritin	1.001	1.000	1.002	**0.001**
Systolic blood pressure	0.970	0.936	1.006	**0.097**
Diastolic blood pressure	0.940	0.881	1.002	**0.058**
Peritonitis rate	1.729	1.218	2.454	**0.002**
Dialysis duration	1.007	0.995	1.018	0.255
Duration of chronic kidney disease	0.791	0.665	0.942	**0.008**
Index of physical impairment (IPI)	2.853	1.427	5.705	**0.003**
Index of coexistent disease (ICED)	2.488	1.064	5.820	**0.035**
Residual urine	0.457	0.183	1.140	**0.093**
Ultrafiltration	1.121	0.426	2.949	0.817
GNL signal	0.042	0.993	1.111	**0.087**

**Table 4:** Results of univariate Cox regression analysis after sorting patients based on their primary disease in correlation with data obtained from lectin microarray with GNL between survivors (*n* = 39) and non-survivors (*n* = 13).

	Patients, *n*	Diseased, *n*	Hazard Ratio	95% Confidence Interval		*p*-Value
				Lower	Upper	
Diabetes mellitus	16	4	0.805	0.256	2.529	0.710
Arterial hypertension	13	3	1.592	0.542	4.672	0.397
Glomerulonephritis	11	3	0.884	0.249	3.133	0.848
Autosomal dominant polycystic kidney disease	4	1	0.731	0.096	5.568	0.763
Tubulointerstitial nephritis	6	2	1.158	0.261	5.135	0.847
Obsturctive uropathy	2	-	0.048	0.000	184,480.792	0.695

**Table 5:** Multivariate Cox regression analysis for survival rate after an 18-month period: survivors (*n* = 39) and non-survivors (*n* = 13). Statistical differences are given in bold.

	Hazard Ratio	95% Confidence Interval		*p*-Value
		Lower	Upper	
Age	1.062	0.985	1.144	0.115
Albumin	0.991	0.833	1.177	0.914
Duration of chronic kidney disease	0.011	0.000	44.582	0.289
Peritonitis rate	0.825	0.462	1.475	0.517
index of coexisting disease (ICED)	1.784	0.338	9.410	0.495
Urea clearance (Kt/V)	0.750	0.180	3.116	0.692
Glucose	1.144	1.008	1.297	**0.037**
Ferritin	1.001	1.000	1.002	0.119
Systolic blood pressure	0.992	0.940	1.047	0.781
Residual urine	3.971	0.626	25.192	0.144
GNL signal	1.100	1.023	1.183	**0.010**

## Data Availability

Not applicable.
